# Interparental and Intergenerational Co-parenting Conflict and Adolescent Academic Performance: The Mediating Roles of Adolescent Academic Engagement and Depression

**DOI:** 10.3390/ijerph192315952

**Published:** 2022-11-30

**Authors:** Hexin Yang, Chaoyue Wu, Ji-Kang Chen

**Affiliations:** 1 Department of Social Work, Wuhan University, Wuhan 430072, China; 2 Department of Social Welfare, University of California, Los Angeles, Los Angeles, CA 90095, USA; 3 Department of Social Work, Chinese University of Hong Kong, Hong Kong, China

**Keywords:** co-parenting, family conflict, academic engagement, depression, academic performance

## Abstract

While a link between co-parenting conflict and academic performance is frequently assumed, studies on this association have shown inconsistent results. In addition, academic engagement and depression can potentially mediate the association between co-parenting conflict and academic performance. However, studies have not tested this proposition. This paper examined the direct effect of co-parenting conflict on adolescent academic performance and the mediating effect of academic engagement and depression. Using data from a nationally representative survey, the 2020 China Family Panel Studies (CFPS), we constructed a sample of 1989 dyads of adolescents (aged 10 to 15) and their primary caregivers in China. The structural equation model analysis revealed that co-parenting conflict was not directly linked with academic performance but was indirectly associated with adolescent academic performance through academic engagement and depression. The findings provide empirical support that academic engagement and depression play important mediating roles in the relationship between co-parenting conflict and adolescent academic performance. Future intervention programs aimed at promoting adolescent academic performance may consider a family-oriented approach to identify adolescents from families with co-parenting conflict and provide them with professional support.

## 1. Introduction

Academic performance is an important factor in adolescent development [1]. Previous research has suggested that students’ academic performance has long-term effects on future academic and career development, and on behavioral and psychological wellbeing [2,3,4,5,6,7]. Better academic performance in adolescence predicts students’ academic success at higher levels of education [6], higher future salaries [5], decreased drug use in midlife [2], and fewer psychiatric symptoms in mid-adulthood [3].

In the past few decades, many studies have explored the effects of family factors on academic performance [8,9,10,11]. Many theories have emphasized that among these family factors, co-parenting conflict, which refers to disagreements and conflicts between the main caregivers over childrearing issues [12], should be one of the most important factors leading to adolescents’ academic performance [13]. For example, family system theory suggests that co-parenting conflict undermines the sense of predictability, stability, and security and creates confusion and hostility in the family context, which might be detrimental to adolescent development, including academic outcomes [13,14,15]. However, previous empirical studies on the association between co-parenting conflict and academic achievement presented inconsistent results. Some studies showed strong relationships between them [16,17], and others showed weak or non-significant associations [9,15,18]. Such inconsistent results may imply that certain psychosocial mechanisms mediate the association between co-parenting conflict and academic performance [19,20,21,22].

After reviewing the literature, we further argue that individual student factors, particularly academic/school engagement, and internalizing problems, such as depression, could mediate the association between co-parenting conflict and academic performance [23,24,25]. The direct effects of co-parenting conflict on academic performance may be mediated through adolescents’ academic engagement and depression. 

Several theories and frameworks support our argument. For example, a theoretical framework proposed by Lui et al. [23] illustrated how adolescents’ family and individual factors interact with each other to contribute to adolescents’ academic performance. Lui et al. [23] argued that adolescents’ family and individual factors may directly influence academic performance in this framework. They further argued that family factors should affect academic performance through individual factors as mediators. The co-parenting conflict has been considered to be an important family factor [9,13,14,15,16], and adolescent academic engagement [26,27,28,29,30] and depression [31,32,33] have been widely recognized as major individual factors in the literature that influence academic performance. Accordingly, it is proposed that co-parenting conflict may influence academic performance via adolescents’ school engagement and depression as mediators. 

Other theories also strengthen our proposition. For example, the resource-allocation model hypothesizes that an individual’s cognitive resources are limited [34]. Once adolescents simultaneously encounter different challenges, they usually need to allocate cognitive resources to deal with each task, which may reduce their efficiency in completing the major one [34,35]. Co-parenting conflict is one of the major influencers in adolescents’ life distracting and reducing their engagement in academic activities [13,36,37]. In addition, previous studies have shown that academic engagement, such as exhibiting motivation to learn, concentrating on learning, exerting effort, and persistence, is an influential factor in academic success [26,38]. Therefore, once adolescents encounter co-parenting conflict in the family, their level of concentration or engagement in school activities may be reduced [13,36], which in turn negatively influences their academic performance [29]. In addition, emotional security theory posits that family conflict provokes adolescent insecurity or negative emotional reactivity, such as depression, undermining their developmental outcomes and academic achievement [39,40,41,42]. Co-parenting conflict is a common family conflict that increases students’ psychological distress [13,37,43,44]. Accordingly, adolescents experiencing co-parenting conflict are prone to emotional insecurity and become depressed [37], which may adversely affect their academic performance [33]. To the best of our knowledge, empirical studies on the indirect association of co-parenting conflict with academic performance through academic engagement and depression are lacking. As a result, how co-parenting conflict, academic engagement, and depression work together to influence adolescents’ academic performance is still unknown. 

Furthermore, most previous studies on the association between co-parenting conflict and academic performance have solely focused on the co-parenting conflict between parents and paid relatively little attention to other major caregivers, such as adolescents’ grandparents, who also play an important role in child rearing in many countries, particularly in Chinese cultural context [13,14,45]. In China, under the influence of familism, family connectedness and obligation fulfillment are highly valued [46]. Even after they start a family and have their own children, Chinese adult children still rely on their parents to some extent [47]. Seniors often show a strong willingness and responsibility to support their adult children, including rearing their grandchildren [48]. Unlike most western grandparents who do not interfere with childrearing, Chinese grandparents view childrearing as a joint mission between them and their adult children [49]. In addition to cultural norms, socioeconomic factors also contribute to the involvement of Chinese grandparents in parenting [46]. 

Given that China has one of the highest rates of female employment [47], the emergence of dual-earner families leaves young parents with little time and energy to take care of their children [50]. Therefore, intergenerational co-parenting is common in China [48,51]. For example, surveys showed that the rate of grandparents participating in childrearing ranges from around 40% in urban China to 90% in some rural areas [50,51,52]. Chinese grandparents share the caregiving responsibility with their adult children and are involved in many aspects of childrearing practices, such as looking after grandchildren, educating and disciplining them, and providing parenting advice to their adult children [47,49]. The two generations’ different childrearing philosophies and methods may trigger co-parenting conflicts between parents and grandparents [47]. In this case, intergenerational parenting conflict could be an important component of co-parenting conflict, which may impede adolescent development [12,15,49].

The current study explored the relationship between co-parenting conflict and academic performance, focusing on the mediating roles of academic engagement and depression. It used a nationally representative random sample and multi-information data from 10–15 years-old adolescents and their primary caregivers in mainland China. 

**Figure 1:**
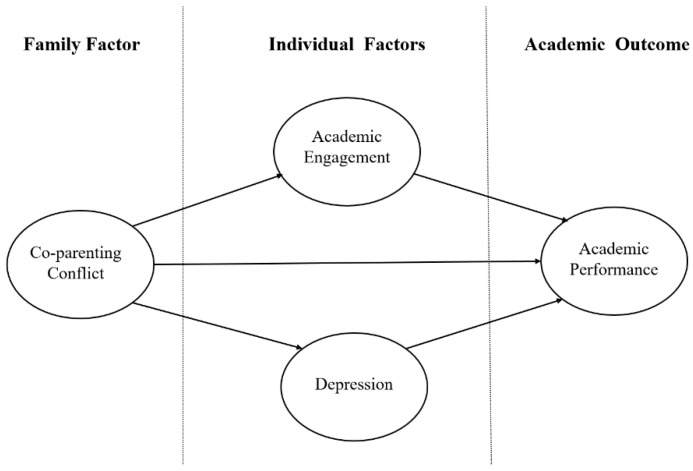
Hypothesized model.

Based on the theories and empirical studies discussed above, this study proposed that children from families with more co-parenting conflicts are more likely to report higher levels of depression, less academic engagement, and worse academic performance. In particular, this study hypothesized that higher levels of depression and less academic engagement would mediate the relationship between co-parenting conflict and academic performance. We proposed that adolescents from families with higher levels of co-parenting conflict were more likely to report higher levels of depression and less academic engagement, thereby undermining their academic performance (see Figure 1).

## 2. Methods

### 2.1. Data and Sampling

The data used in this study were from the sixth round of China Family Panel Studies (CFPS) conducted in 2020. CFPS is a nationwide longitudinal survey of communities, households, and individuals in mainland China, assessing topics such as family dynamics, mental health, and education outcomes [53]. A three-stage probability sampling strategy was adopted. In the first stage, 162 county-level units were selected from 25 provinces, representing 95% of the Chinese population. In the second stage, two or four communities were chosen in each county. Socioeconomic indicators, such as GDP and population size, were used for implicit stratification in the first two stages. In the third stage, households in those communities were selected using systematic sampling [54].

Written consent was obtained from all participants before the formal survey. The survey was conducted via face-to-face interview or telephone interview from July 2020 to December 2020. Further detailed information on informed consent and ethical concerns can be found on the official website of CFPS (http://www.isss.pku.edu.cn/cfps/, accessed on 26 November 2022).

The sample included 10–15 years old adolescents attending elementary and middle schools, as self-reported in the 2020 CFPS questionnaire (*n* = 2143). Overall, 7.19% (*n* = 154) of the adolescents whose caregivers did not complete corresponding questionnaires were excluded from this study. The final sample consisted of 1989 parent and child dyads. Of the children, 938 (47.16%) were girls and 1051 (52.84%) children were boys.

### 2.2. Measurement

#### 2.2.1. Academic Performance

Three items measured this latent variable. One item asked adolescents to report their academic rank in class at the latest examination on a five-point Likert scale (1 = the top 10%, 2 = 11%–25%, 3 = 26%–50%, 4 = 51%–75%, 5 = the bottom 24%), with a factor loading of 0.569. Another two items asked the primary caregivers of adolescents to report their child’s grade in two subjects (Chinese and math) last semester on a four-point Likert scale (1 = excellent to 4 = poor). The factor loading was 0.837 for the Chinese and 0.797 for the math grades. Scores on these three items were recoded (1 = the bottom 24%, 2 = 51%–75%, 3 = 26%–50%, 4 = 11%–25%, 5 = the top 10%) (1 = poor to 4 = excellent) so that higher scores indicated better academic performance. The Cronbach’s alpha of these three items was 0.746.

#### 2.2.2. Co-parenting Conflict

This latent variable was measured by two items asking the adolescents’ primary caregivers about the frequency of family conflict about parenting in the past 12 months. A five-point Likert scale was used (1 = never to 5 = very often). The two items were: “How often did the parents and the grandparents of the adolescents disagree over childrearing?”; and “How often did parents disagree with each other over childrearing?”. The factor loadings were 0.935 and 0.793, respectively. The Cronbach’s alpha of these two items was 0.709.

#### 2.2.3. Academic Engagement

Academic engagement was measured by five items asking the primary caregivers of adolescents to evaluate their child’s engagement in academic tasks on a five-point Likert scale (1 = strongly disagree to 5 = strongly agree). These five items were: “Your child studies very hard” (factor loading =0.754); “Your child checks homework for errors after completing it” (factor loading =0.805); “Your child won’t play until finishing homework” (factor loading = 0.721); “Your child is attentive during class-time” (factor loading = 0.791); and “Your child will complete what he started” (factor loading = 0.691). The Cronbach’s alpha of these five items was 0.802.

#### 2.2.4. Depression

Depression was measured by six items asking the adolescents about their depressive symptoms during the past week. All items were selected from a depression scale included in the Center for Epidemiological Studies-Depression (CES-D) [55,56] and measured on a four-point Likert scale (1 = never to 4 = most of the time). These six items were: “I felt depressed” (factor loading = 0.703); “I felt that everything I did was an effort” (factor loading = 0.684); “My sleep was restless” (factor loading = 0.658); “I felt lonely” (factor loading = 0.783); “I felt sad” (factor loading = 0.773); and “I could not get going’” (factor loading = 0.770). The Cronbach’s alpha of these six items was 0.781. 

### 2.3. Data Analysis Plan

Descriptive statistics (the means and standard deviations) and a correlation matrix of all variables were computed with SPSS Version 24.0 (IBM, Armonk, NY, USA). Subsequently, Mplus Version 8.3 statistical software (Muthén & Muthén, Los Angeles, CA, USA) was employed to examine the proposed theoretical model of this study. The weighted least squares with mean and variance adjusted (WLSMV) estimator was used because the data were ordinal [57]. A latent variables structural equation modeling (SEM) was conducted, using the bootstrapping approach (*n* = 5000 bootstrap samples) [58]. 

The comparative fit index (CFI), the Tucker-Lewis index (TLI), the root mean square error of approximation (RMSEA), and the standardized root mean squared residual (SRMR) were adopted as model fit indices in this study. Typically, CFI and TLI above 0.95 [59,60], and RMSEA and SRMR below 0.05 [61] indicate a good model fit. 

The full information maximum likelihood (FIML) was used to address missing values.

## 3. Results

### 3.1. Descriptive Statistics

Table 1 shows the mean and standard deviations of each variable in this study by gender. The correlations between all variables are displayed in Table 2. The results indicated that academic performance was negatively correlated with co-parenting conflict (*r* = −0.069, *p* < 0.01) and depression (*r* = −0.127, *p* < 0.001), and positively related to academic engagement (*r* = 0.253, *p* < 0.001). Co-parenting conflict negatively with academic engagement (*r* = −0.125, *p* < 0.001) and positively correlated with depression (*r* = 0.121, *p* < 0.001). Depression and academic engagement were negatively correlated (*r* = −0.047, *p* < 0.05).

### 3.2. The Overall Model

The results of SEM indicated that the theoretical model fit the data well, χ^2^ (98, *N* = 1989) = 220.171, *p* < 0.001, RMSEA = 0.025, CFI = 0.993, TLI = 0.992, SRMR = 0.025. 

**Figure 2:**
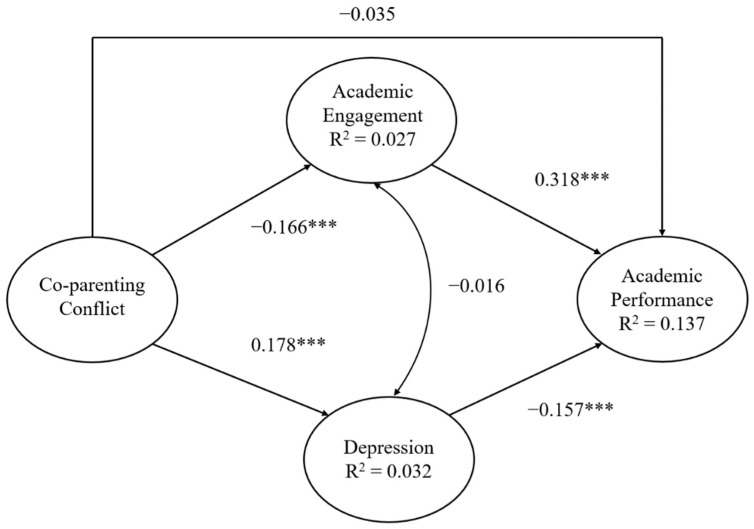
Structural equation model of direct and indirect effects on academic performance for Chinese 10–15-year-old adolescents. *** *p* < 0.001.

Figure 2 shows that the co-parenting conflict had significant negative associations with academic engagement (*β* = −0.166, *p* < 0.001) and significant positive associations with depression (*β* = 0.178, *p* < 0.001). Academic performance was significantly and directly associated with academic engagement (*β* = 0.318, *p* < 0.001) and depression (*β* = −0.157, *p* < 0.001). Although the co-parenting conflict was not directly associated with academic performance (*β* = −0.035, *p* > 0.05), the indirect effects of academic performance and co-parenting conflict via academic engagement and depression were significant.

We randomly generated 5000 bootstrapping samples from the original dataset to assess the mediating effects of academic engagement and depression on co-parenting conflict and academic performance. The results revealed that the indirect effects of co-parenting conflicts on academic performance through academic engagement and depression were, respectively, −0.053 (SE = 0.012, CI = [−0.079, −0.031]) and −0.028 (SE = 0.008, CI = [−0.046, −0.015]). The 95% confidence interval did not contain zero, confirming that co-parenting conflict significantly affected academic performance via academic engagement and depression. 

The overall model accounted for 13.7% of the explained variance in academic performance (R^2^ = 0.137).

## 4. Discussion

### 4.1. The Overall Model

Using a national representative random sample from mainland China, this study examined mediating effects of individual factors (i.e., adolescent academic engagement and depression) on the association between a family factor (i.e., co-parenting conflicts) and academic performance. In addition, unlike most previous studies in western countries measuring the co-parenting conflict among parents, this study included conflicts between parents and other major caregivers (i.e., grandparents). The results of this study showed that our proposed model had a good fit to the data, suggesting that the mediation effect of academic engagement and depression on the association between co-parenting conflict and academic performance applies to adolescents aged 10–15 years in the Chinese cultural context.

The direct association between co-parenting conflict and academic performance was insignificant, which is in line with empirical studies showing a weak or insignificant link between co-parenting conflict and its outcome on adolescents’ academic performance [9,15,18]. 

However, the results of this study showed that co-parenting conflict indirectly affects academic performance through academic engagement and depression. That is, adolescents from families with higher levels of co-parenting conflict are more likely to reduce their engagement in educational activities and suffer from depressive symptoms, which may decrease their academic performance. These findings support our theoretical model positing that adolescent academic engagement and depression mediate the relationships between co-parenting conflict and academic performance. The findings provide evidence that co-parenting conflict may lead to inconsistent parenting discipline practices in families, making children confused and stressed, unable to decide which caregiver to affiliate with and which directives to follow [62]. This process might strain cognitive resources and distract children’s attention from learning, which is in line with resource-allocation theory [13,34]. Once their involvement in academic activities decreases, they are less likely to integrate new information with existing knowledge and form more complex knowledge structures, negatively affecting their academic performance [26,30,63]. In addition, our findings support emotional security theory, which explains that adolescents who encounter family conflict, such as co-parenting conflict, tend to have feelings of insecurity and depressive symptoms, which are harmful to their overall development [39,41]. 

These results are also consistent with the framework proposed by Lui et al. [23], which suggests the indirect pathways from family factors to adolescents’ academic performance through individual factors. Certain psychosocial mechanisms, such as individual behavioral and emotional responses to co-parenting conflict, influence the level of academic performance rather than the co-parenting conflict itself. Compared with co-parenting conflict, adolescent academic engagement and depressive symptoms had a greater influence on academic performance.

In addition, this study indicated that academic engagement plays a stronger mediating role and accounts for a larger amount of the explained variance in academic performance than depression. This is in line with previous studies suggesting that academic engagement has a more salient effect than depression on student academic performance [64]. These findings imply that a higher level of academic engagement is a robust factor leading to better academic performance among students in early adolescence in mainland China. 

### 4.2. Limitations

Some limitations need to be considered when interpreting the results of this study. First, this study used cross-sectional data; therefore, we cannot make causal links among variables. Future research may utilize longitudinal data to prove the causality. Second, this study relied on a random sample of adolescents aged 10 to 15 years in mainland China. The results may not be generalizable to adolescents in different age groups or cultural contexts. Third, only two items were used to measure the frequency of co-parenting conflict variable in this study. The co-parenting conflict is a multifaceted concept [65]. Future research may consider developing more valid intergenerational co-parenting scales and interparental co-parenting scales including other characteristics of co-parenting conflict, such as covert conflict or overt conflict, to comprehensively understand this issue. We also recommend that future research should further examine the independent influences of intergenerational and interparental co-parenting conflict on adolescent psychological, behavioral, and academic outcomes based on the current study.

### 4.3. Implications

Despite its limitation, this study extends the existing literature by using a nationally random sample of Chinese adolescents and showing that co-parenting conflicts, academic engagement, and depression account for a significant amount of the explained variance in academic performance. Our findings emphasize the harmful effect of co-parenting conflict on children’s academic outcomes. A family-oriented intervention should encourage harmonious co-parenting relationships, improve co-parents’ capacity to handle childrearing disagreements and conflicts, and reduce co-parenting conflict [66].

Our findings also highlight the mediating roles of academic engagement and depression in the relationship between co-parenting conflict and academic performance. In addition, compared to depression, academic engagement has a much stronger effect on academic performance. Interventions aiming to promote adolescent academic performance should focus more on adolescents from families with heightened levels of co-parenting conflict. Subsequently, practitioners could provide these adolescents with professional support through depression interventions designed to reduce their depression levels and school engagement programs designed to promote their engagement in academic activities. These strategies might alleviate the negative effects of co-parenting conflict on adolescents’ academic outcomes and improve their academic performance. 

## 5. Conclusions

In summary, our study empirically supports the indirect relationship of co-parenting conflict with academic performance through academic engagement and depression as mediators. Hence, a family-oriented approach may effectively advance the academic performance of adolescents experiencing co-parenting conflict. The research findings indicate adolescent academic engagement and depression play important mediating roles in the association between co-parenting conflicts and academic performance. Future interventions could focus on promoting adolescent academic engagement and decreasing their depressive symptoms to improve their academic performance. Such programs may be more effective when combined with co-parenting conflict interventions. 

## Figures and Tables

**Table 1:** Means and standard deviations of variables for the whole sample and both gender groups (standard deviations in parenthesis).

	Total	Boys	Girls
Academic performance ^a^	8.918 (2.437)	9.037 (2.410)	8.784 (2.461)
Co-parenting conflict ^b^	3.068 (1.565)	3.065 (1.565)	3.071 (1.565)
Academic engagement ^c^	17.781 (3.765)	17.781 (3.752)	17.781 (3.781)
Depression ^d^	8.718 (2.888)	8.673 (2.840)	8.769 (2.942)

Note. The minimum and maximum values of the variables are as follows: ^a^ academic performance: from 3 to 13; ^b^ co-parenting conflict: from 2 to 10; ^c^ academic engagement: from 5 to 25; ^d^ depression: from 6 to 24.

**Table 2:** Intercorrelations between all variables.

	1	2	3	4
1. Academic performance	—			
2. Co-parenting conflict	−0.069 **	—		
3. Academic engagement	0.253 ***	−0.125 ***	—	
4. Depression	−0.127 ***	0.121 ***	−0.047 *	—

Note. * *p* < 0.05; ** *p* < 0.01; *** *p* < 0.001.

## Data Availability

The data used in this study are available on the CFPS website (www.isss.pku.edu.cn/cfps/, accessed on 26 November 2022).

## References

[1] Peng P., Kievit R.A. (2020). The Development of Academic Achievement and Cognitive Abilities: A Bidirectional Perspective. Child Dev. Perspect..

[2] Kendler K.S., Ohlsson H., Fagan A.A., Lichtenstein P., Sundquist J., Sundquist K. (2018). Academic Achievement and Drug Abuse Risk Assessed Using Instrumental Variable Analysis and Co-relative Designs. JAMA Psychiatry.

[3] Pijnenburg L.J., de Haan L., Smith L., Rabinowitz J., Levine S.Z., Reichenberg A., Velthorst E. (2021). Early predictors of mental health in mid-adulthood. Early Interv. Psychiatry.

[4] Robert P.-O., Kuipers M.A.G., Rathmann K., Moor I., Kinnunen J.M., Rimpelä A., Perelman J., Federico B., Richter M., Kunst A.E. (2018). Academic performance and adolescent smoking in 6 European cities: The role of friendship ties. Int. J. Adolesc. Youth.

[5] Rudakov V., Roshchin S. (2019). The impact of student academic achievement on graduate salaries: The case of a leading Russian university. J. Educ. Work..

[6] Saunders-Scott D., Braley M.B., Stennes-Spidahl N. (2017). Traditional and psychological factors associated with academic success: Investigating best predictors of college retention. Motiv. Emot..

[7] Sorberg Wallin A., Koupil I., Gustafsson J.E., Zammit S., Allebeck P., Falkstedt D. (2019). Academic performance, externalizing disorders and depression: 26,000 adolescents followed into adulthood. Soc. Psychiatry Psychiatr. Epidemiol..

[8] Harold G.T., Sellers R. (2018). Annual Research Review: Interparental conflict and youth psychopathology: An evidence review and practice focused update. J. Child Psychol. Psychiatry.

[9] Ren L., Cheung R.Y.M., Boise C., Li X., Fan J. (2020). Fathers’ perceived co-parenting and children’s academic readiness among Chinese preschoolers: Longitudinal pathways through parenting and behavioral regulation. Early Child. Res. Q..

[10] Zhang C. (2019). Are Children from Divorced Single-Parent Families Disadvantaged? New Evidence from the China Family Panel Studies. Chin. Sociol. Rev..

[11] Zhang X., Hu B.Y., Ren L., Huo S., Wang M. (2019). Young Chinese Children’s Academic Skill Development: Identifying Child-, Family-, and School-Level Factors. New Dir. Child Adolesc. Dev..

[12] Feinberg M.E., Kan M.L., Hetherington E.M. (2007). The longitudinal influence of coparenting conflict on parental negativity and adolescent maladjustment. J. Marriage Fam..

[13] Boričević Maršanić V., Kušmić E. (2013). Coparenting within the family system: Review of literature. Coll. Antropol..

[14] Feinberg M.E. (2003). The Internal Structure and Ecological Context of Coparenting: A Framework for Research and Intervention. Parent. Sci. Pract..

[15] Jahromi L.B., Zeiders K.H., Updegraff K.A., Umana-Taylor A.J., Bayless S.D. (2018). Coparenting Conflict and Academic Readiness in Children of Teen Mothers: Effortful Control as a Mediator. Fam. Process.

[16] Cabrera N.J., Scott M., Fagan J., Steward-Streng N., Chien N. (2012). Coparenting and children’s school readiness: A mediational model. Fam. Process.

[17] Pendry P., Adam E.K. (2013). Child-Related Interparental Conflict in Infancy Predicts Child Cognitive Functioning in a Nationally Representative Sample. J. Child Fam. Stud..

[18] McHale J.P., Rao N., Krasnow A.D. (2000). Constructing family climates: Chinese mothers’ reports of their co-parenting behaviour and preschoolers’ adaptation. Int. J. Behav. Dev..

[19] Chen J.K., Wu C., Chang C.W., Wei H.S. (2020). Indirect effect of parental depression on school victimization through adolescent depression. J. Affect. Disord..

[20] Chen J.K., Pan Z., Wang L.C. (2021). Parental Beliefs and Actual Use of Corporal Punishment, School Violence and Bullying, and Depression in Early Adolescence. Int. J. Environ. Res. Public Health.

[21] Chen J.-K., Wang S.-C., Chen Y.-W., Huang T.-H. (2021). Family Climate, Social Relationships With Peers and Teachers at School, and School Bullying Victimization Among Third Grade Students in Elementary Schools in Taiwan. Sch. Ment. Health.

[22] Chen J.-K., Wang Z., Wong H., Tang V.M.-Y. (2021). Child Deprivation as a Mediator of the Relationships between Family Poverty, Bullying Victimization, and Psychological Distress. Child Indic. Res..

[23] Lui M., Lau G.K., Tam V.C., Chiu H.-M., Li S.S., Sin K.-F. (2019). Parents’ Impact on Children’s School Performance: Marital Satisfaction, Parental Involvement, and Mental Health. J. Child Fam. Stud..

[24] Shehni-Yailagh M., Azizimehr A., Maktabi G. (2014). The causal relationship between covert and overt conflicts and school performance, mediated by parent-child relationship, antisocial behavior and withdrawal/depression among high school students. Biannu. J. Appl. Couns..

[25] Swanson J., Valiente C., Lemery-Chalfant K. (2012). Predicting Academic Achievement from Cumulative Home Risk: The Mediating Roles of Effortful Control, Academic Relationships, and School Avoidance. Merrill-Palmer Q..

[26] Alrashidi O., Phan H.P., Ngu B.H. (2016). Academic Engagement: An Overview of Its Definitions, Dimensions, and Major Conceptualisations. Int. Educ. Stud..

[27] Dogan U. (2015). Student Engagement, Academic Self-efficacy, and Academic Motivation as Predictors of Academic Performance. Anthropologist.

[28] Gunuc S. (2014). The relationships between student engagement and their academic achievement. Int. J. New Trends Educ. Implic..

[29] Lee J.-S. (2014). The Relationship Between Student Engagement and Academic Performance: Is It a Myth or Reality?. J. Educ. Res..

[30] Lei H., Cui Y., Zhou W. (2018). Relationships between student engagement and academic achievement: A meta-analysis. Soc. Behav. Personal. Int. J..

[31] Khesht-Masjedi M.F., Shokrgozar S., Abdollahi E., Habibi B., Asghari T., Ofoghi R.S., Pazhooman S. (2019). The relationship between gender, age, anxiety, depression, and academic achievement among teenagers. J. Fam. Med. Prim. Care.

[32] Shen W. (2020). A tangled web: The reciprocal relationship between depression and educational outcomes in China. Soc. Sci. Res..

[33] Wickersham A., Sugg H.V.R., Epstein S., Stewart R., Ford T., Downs J. (2021). Systematic Review and Meta-analysis: The Association Between Child and Adolescent Depression and Later Educational Attainment. J. Am. Acad. Child Adolesc. Psychiatry.

[34] Zemp M., Bodenmann G., Mark Cummings E. (2014). The role of skin conductance level reactivity in the impact of children’s exposure to interparental conflict on their attention performance. J. Exp. Child Psychol..

[35] Schneider W., Fisk A.D. (1982). Concurrent automatic and controlled visual search: Can processing occur without resource cost?. J. Exp. Psychol. Learn. Mem. Cogn..

[36] Dopkins Stright A., Neitzel C. (2003). Beyond parenting: Coparenting and children’s classroom adjustment. Int. J. Behav. Dev..

[37] Teubert D., Pinquart M. (2010). The Association Between Coparenting and Child Adjustment: A Meta-Analysis. Parenting.

[38] Finn J.D., Zimmer K.S. (2012). Student Engagement: What Is It? Why Does It Matter?. Handbook of Research on Student Engagement.

[39] Cummings E.M., Davies P.T. (2002). Effects of marital conflict on children: Recent advances and emerging themes in process-oriented research. J. Child Psychol. Psychiatry.

[40] Cummings E.M., Miller-Graff L.E. (2015). Emotional Security Theory: An Emerging Theoretical Model for Youths’ Psychological and Physiological Responses Across Multiple Developmental Contexts. Curr. Dir. Psychol. Sci..

[41] Davies P.T., Martin M.J., Sturge-Apple M.L. (2016). Emotional security theory and developmental psychopathology. Dev. Psychopathol..

[42] Xiang X., Wang J., Wu C., Chen Y. (2022). Interparental Violence and Early Adolescents’ Adjustment Problems in China: Testing a Moderated Mediation Model of Parental Warmth and Emotional Insecurity. J. Interpers. Violence.

[43] Rejaän Z., van der Valk I.E., Branje S. (2021). Postdivorce Coparenting Patterns and Relations With Adolescent Adjustment. J. Fam. Issues.

[44] Zou S., Wu X., Huang B., Liu C. (2019). Double-Edged Effect of Coparenting on Chinese Adolescents’ Emotional Instability: An Inconsistent Mediation Model. J. Child Fam. Stud..

[45] Liang X., Lin Y., Van I.M.H., Wang Z. (2021). Grandmothers are part of the parenting network, too! A longitudinal study on coparenting, maternal sensitivity, child attachment and behavior problems in a Chinese sample. New Dir. Child Adolesc. Dev..

[46] Wang C.D., Hayslip B., Sun Q., Zhu W. (2019). Grandparents as the Primary Care Providers for Their Grandchildren: A Cross-Cultural Comparison of Chinese and U.S. Samples. Int. J. Aging Hum. Dev..

[47] Goh E.C.L., Kuczynski L. (2010). ‘Only children’ and their coalition of parents: Considering grandparents and parents as joint caregivers in urban Xiamen, China. Asian J. Soc. Psychol..

[48] Li X., Liu Q. (2020). Parent–grandparent coparenting relationship, marital conflict and parent–child relationship in Chinese parent–grandparent coparenting families. Child. Youth Serv. Rev..

[49] Tai T.-O., Tu H.-C. (2021). The Effect of Grandparental Care on Men’s and Women’s Parenting Practices in Taiwan. J. Popul. Ageing.

[50] Li X., Liu Y. (2019). Parent-Grandparent Coparenting Relationship, Maternal Parenting Self-efficacy, and Young Children’s Social Competence in Chinese Urban Families. J. Child Fam. Stud..

[51] Luo N., Van Heel M., Van Leeuwen K. (2020). Perspectives of Early Adolescents, Parents, and Grandparents on Parenting Behaviors in China. J. Early Adolesc..

[52] Sun J. (2012). Chinese Older Adults Taking Care of Grandchildren: Practices and Policies for Productive Aging. Ageing Int..

[53] Xie Y., Hu J. (2014). An introduction to the China family panel studies (CFPS). Chin. Sociol. Rev..

[54] Xie Y., Lu P. (2015). The Sampling Design of the China Family Panel Studies (CFPS). Chin. J. Sociol..

[55] Radloff L.S. (1977). The CES-D scale: A self-report depression scale for research in the general population. Appl. Psychol. Meas..

[56] Zhou X., Sun X. (2021). Family depression profiles among adolescents and their parents: A group-based multitrajectory modeling. J. Fam. Psychol..

[57] Muthén L.K., Muthén B.O. (2010). Mplus User’s Guide: Statistical Analysis with Latent Variables: User’s Guide.

[58] Preacher K.J., Hayes A.F. (2008). Asymptotic and resampling strategies for assessing and comparing indirect effects in multiple mediator models. Behav. Res. Methods.

[59] Bentler P.M. (1990). Comparative fit indexes in structural models. Psychol. Bull..

[60] Hu L.T., Bentler P.M. (1999). Cutoff criteria for fit indexes in covariance structure analysis: Conventional criteria versus new alternatives. Struct. Equ. Model. A Multidiscip. J..

[61] Schermelleh-Engel K., Moosbrugger H., Müller H. (2003). Evaluating the fit of structural equation models: Tests of significance and descriptive goodness-of-fit measures. Methods Psychol. Res. Online.

[62] Umemura T., Christopher C., Mann T., Jacobvitz D., Hazen N. (2015). Coparenting Problems with Toddlers Predict Children’s Symptoms of Psychological Problems at Age 7. Child Psychiatry Hum. Dev..

[63] Xie K., Vongkulluksn V.W., Lu L., Cheng S.-L. (2020). A person-centered approach to examining high-school students’ motivation, engagement and academic performance. Contemp. Educ. Psychol..

[64] Fiorilli C., De Stasio S., Di Chiacchio C., Pepe A., Salmela-Aro K. (2017). School burnout, depressive symptoms and engagement: Their combined effect on student achievement. Int. J. Educ. Res..

[65] Ferraro A.J., Lucier-Greer M., Oehme K. (2018). Psychometric Evaluation of the Multidimensional Co-Parenting Scale for Dissolved Relationships. J. Child Fam. Stud..

[66] Eira Nunes C., Roten Y., El Ghaziri N., Favez N., Darwiche J. (2020). Co-Parenting Programs: A Systematic Review and Meta-Analysis. Fam. Relat..

